# Pleiotropic functions of *chordin* gene causing drastic morphological changes in ornamental goldfish

**DOI:** 10.1038/s41598-022-24444-7

**Published:** 2022-11-19

**Authors:** Hsiao-Chian Chen, Chenyi Wang, Ing-Jia Li, Gembu Abe, Kinya G. Ota

**Affiliations:** 1 Laboratory of Aquatic Zoology, Marine Research Station, Institute of Cellular and Organismic Biology, Academia Sinica, Yilan, 26242 Taiwan; 2 Division of Developmental Biology, Department of Functional Morphology, Faculty of Medicine, School of Life Science, Tottori University, Nishi-Cho 86, Yonago, 683-8503 Japan

**Keywords:** Developmental biology, Morphogenesis, Evolution, Evolutionary developmental biology

## Abstract

Breeders and fanciers have established many peculiar morphological phenotypes in ornamental goldfish. Among them, the twin-tail and dorsal-finless phenotypes have particularly intrigued early and recent researchers, as equivalent morphologies are extremely rare in nature. These two mutated phenotypes appeared almost simultaneously within a short time frame and were fixed in several strains. However, little is known about how these two different mutations could have co-occurred during such a short time period. Here, we demonstrate that the *chordin* gene, a key factor in dorsal–ventral patterning, is responsible not only for the twin-tail phenotype but also for the dorsal-finless phenotype. Our F2 backcrossing and functional analyses revealed that the penetrance/expressivity of the dorsal-finless phenotype can be suppressed by the wild-type allele of *chdS*. Based on these findings, we propose that *chdS*^*wt*^ may have masked the expression of the dorsal-finless phenotype, acting as a capacitor buffering gene to allow accumulation of genetic mutations. Once this gene lost its original function in the twin-tail goldfish lineages, the dorsal-finless phenotype could be highly expressed. Thus, this study experimentally demonstrates that the rapid genetic fixation of morphological mutations during a short domestication time period may be related to the robustness of embryonic developmental mechanisms.

## Introduction

**Figure 1:**
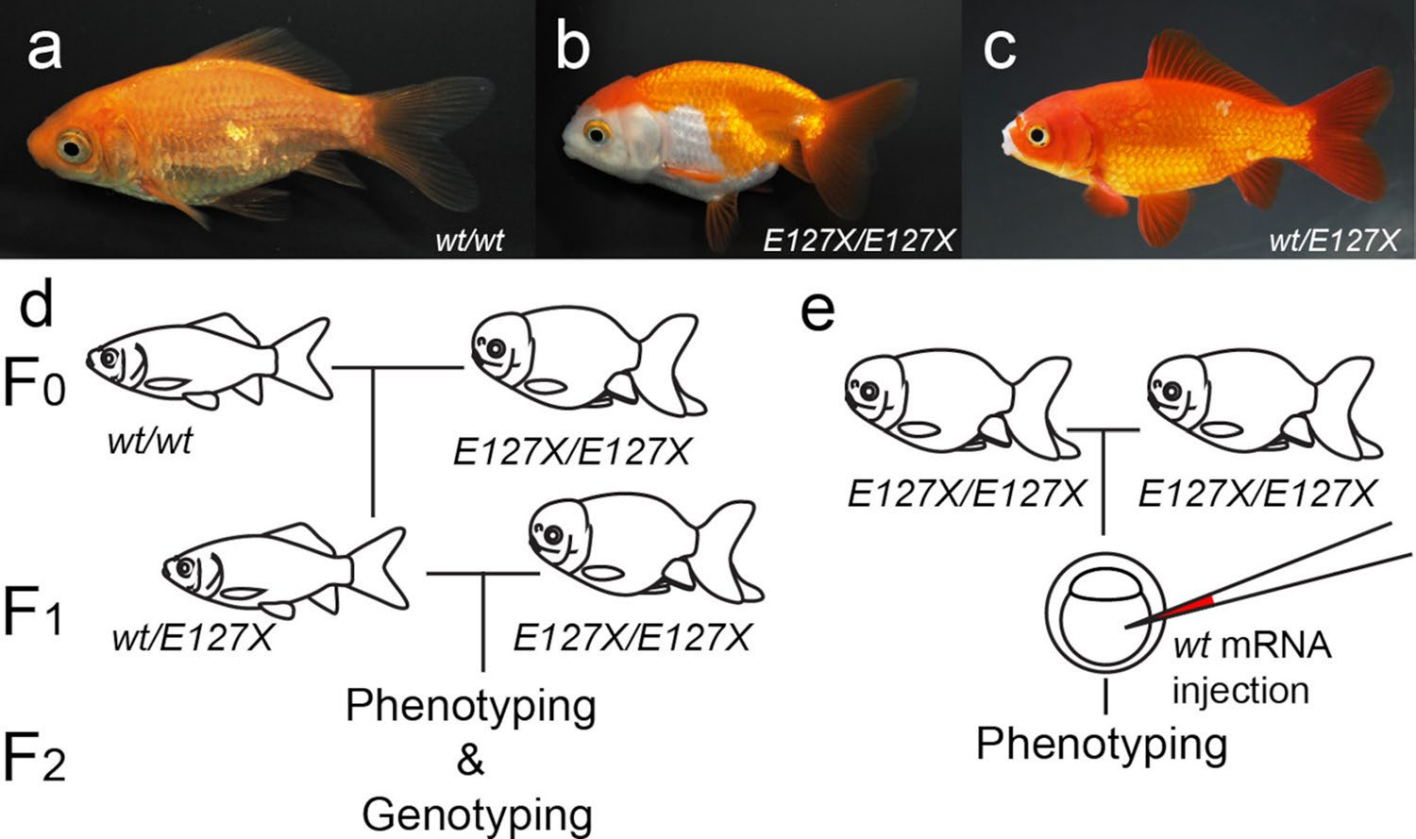
Schematic views of experimental procedures. Lateral views of the wild-type (**a**) and *Ranchu* strain (**b**) goldfish. (**c**) A lateral view of hybrid progeny of *Ranchu* and wild-type. (**d**) Schematic representation of backcross analysis. (**e**) Schematic representation of mRNA microinjection rescue experiment. The size of the individuals in (**a**, **b**, and **c**) are 5 cm, 6 cm and 6 cm approximately, respectively.

Studying the peculiar phenotypes of ornamental animals may provide insights into how animal body shapes are able to change over time. Although domestication processes occur over time scales that are relatively short compared to those required for natural large-scale morphological evolution, breeders and fanciers of dogs, birds and fish have succeeded in establishing many ornamental domesticated animals with highly diverged phenotypes^1–7^. Among these ornamental animals, goldfish (*Carassius auratus*) exhibit especially highly diverged morphological variations in the skeletal system, which are extremely uncommon in naturally selected vertebrate species (Fig. 1a,b)^8–10^. For instance, the dorsal-finless goldfish strains (e.g., *Ranchu*) exhibit bifurcated caudal fins and lack dorsal fins, which have intrigued early and recent researchers due to the rareness of equivalent mutations in other vertebrate linages (Fig. 1b)^6–10^.

While a bifurcated caudal axial skeleton has not been identified in any vertebrate taxa, this morphology is genetically fixed in the twin-tail ornamental goldfish strain^11,12^. The dorsal-finless mutation is also genetically fixed in a domesticated goldfish population as an ornamental variation, but equivalent intra-species variations are not found in teleost species (Fig. 1a,b)^13^. Based on known molecular phylogenetic relationships and surveys of Chinese archives and paintings, these two mutant phenotypes are thought to have quasi-simultaneously appeared and became genetically fixed in the dorsal-finless goldfish strain during a domestication process that lasted less than 600 years (Fig. 1b) (Supplementary Information)^6,8–10,14,15^. It is also revealed that the *chdS* locus (or *chdA* locus in previous study)—the homologue of *chordin* gene which play a significant role for the dorsal–ventral patterning—was identified as the responsible gene of the twin-tail phenotype; a stop codon-containing allele (*chdS*^*E127X*^) is the responsible allele and the *chdS*^*E127X/E127X*^ genotype is commonly shared among all of the investigated ornamental goldfish strains with bifurcated caudal fin, including *Ryukin*, *Oranda*, and *Ranchu*^11^. Subsequently, genome-wide association study (GWAS) methodologies were used to identify multiple candidate loci that might be related with the dorsal-finless phenotype^6,7^. However, little is known about how breeders and fanciers so quickly succeeded to genetically fixing the genes that give rise to such a peculiar ornamental goldfish strain, especially in light of the fact that the morphological mutations occur in multiple complex and sophisticated body parts.

Here, we conducted genetic and functional analyses in the dorsal-finless phenotype focusing on the *chdS* locus. Since all of the investigated modern dorsal-finless goldfish strains share the twin-tail phenotype and *chdS* mutated genotype (*chdS*^*E127X/E127X*^), it is reasonable to suspect that the *chdS* locus may be related not only with the bifurcated caudal fin but also with the dorsal-finless phenotype^6,7,11^. To examine this possibility, we conducted genotyping and phenotyping of backcrossed F2 progenies derived from the hybrids of the single-tail common goldfish (wild-type) and the *Ranchu* strain (Fig. 1a–d)^6,11^. Moreover, we performed microinjection of *chdS*^*wt*^ mRNA into the progenies derived from *Ranchu* strain parents (Fig. 1e). Based on our results, we conclude that the *chdS* gene is indeed responsible for both the twin-tail and dorsal-finless phenotypes, suggesting that these two phenotypes are causally related to each other. These findings provide intriguing insights into how a highly conserved body architecture can be drastically changed during a short time period.

## Results

### Genetic backcross analysis

To conduct our genetic analysis, we generated F2 backcrossed progenies starting with F0 parents of the wild-type and *Ranchu* strain (Fig. 1d; Supplementary Fig. S1). The F0 wild-type and *Ranchu* parents were respectively *chdS*^*wt/wt*^ and *chdS*^*E127X/E127X*^ in their *chdS* genotypes (Fig. 1a,b). This set of F0 wild-type and *Ranchu* individuals yielded a total of 123 F1 progenies in four clutches: 2021-0426-22-RAwt (n = 43), 2018-0328-05 (n = 22), 2021-0510-09 (n = 24), 2021-0510-12 (n = 34). All of these F1 progenies carried a *chdS*^*wt/E127X*^ genotype and exhibited single median fins (including dorsal, anal and caudal fins), similar to the wild-type goldfish (Fig. 1c). Based on this consistent phenotype, we can conclude that the alleles conferred by wild-type goldfish suppress the dorsal-finless phenotype in the F1 progenies.

**Figure 2:**
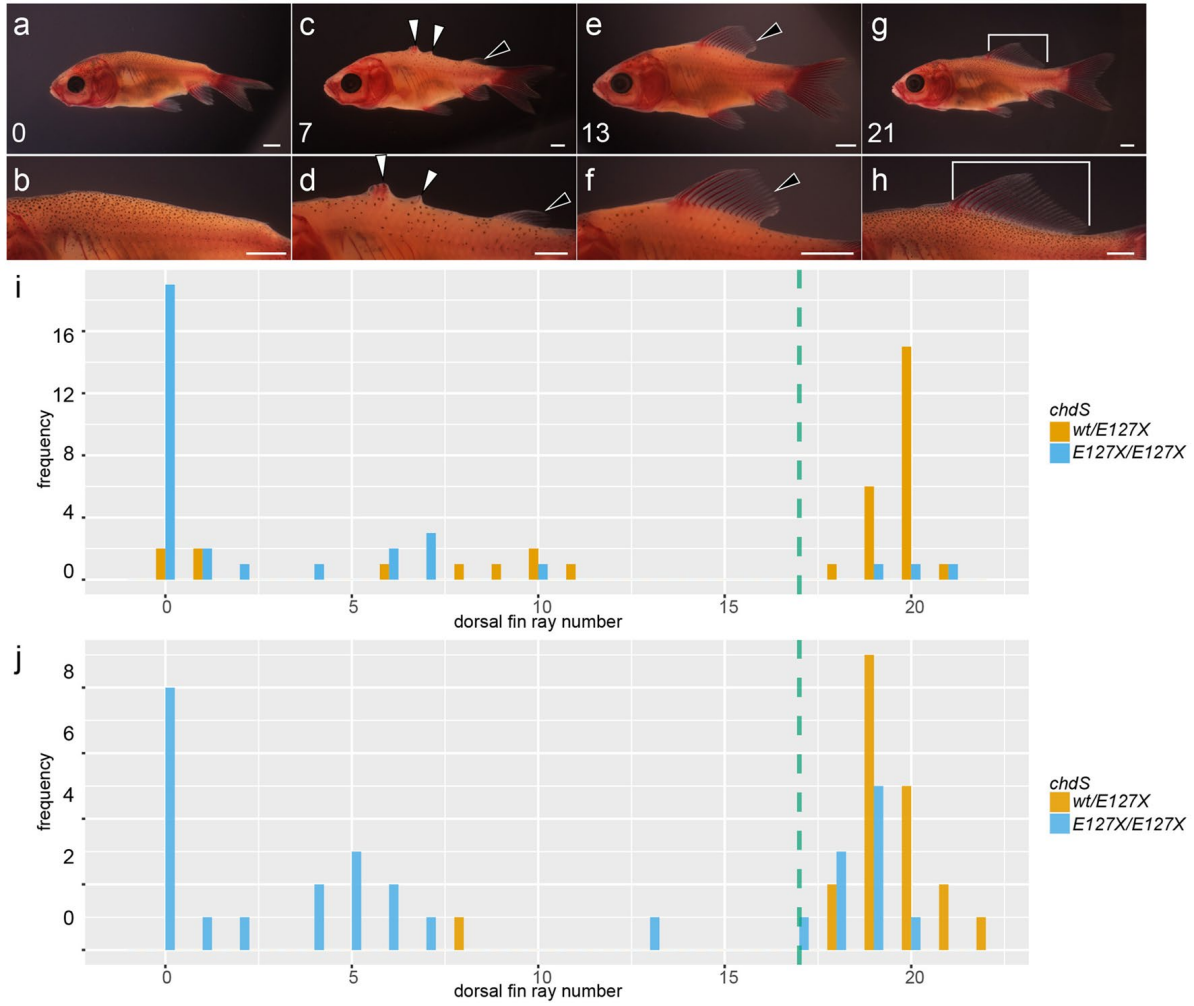
Dorsal fin phenotype in F2 hybrid. (**a**–**h**) Lateral views of the dorsal region [three representative variations of dorsal-finless mutants with bifurcated caudal fins (**a**–**f**) and wildtype (**g**, **h**)]. Numbers in the lower left corner indicate the number of dorsal fin rays including serrated dorsal fin spine and poste-serrated dorsal fin spine rays (please see text in details). Panels of (**b**), (**d**), (**f**), and (**h**) are magnified views of (**a**, **c**, **e**, and **g**), respectively. (**i**, **j)** Histograms of distribution patterns of dorsal fin ray number of *chdS*^*wt/E127X*^ and *chdS*^*E127X/E127X*^ genotypes. (**i**) 2020–0511-06RAWT clutch, (**j**) 2021–0406-01-RAWT clutch). Green-colored dotted vertical line indicates the 17 dorsal fin ray number (the boundary of wild-type and mutant phenotype; see text).

The F1 progenies were then backcrossed with the *Ranchu* strain to generate F2 progenies (Fig. 1d, Supplementary Fig. S1). Two clutches of F2 progenies were obtained and designated “2020-0511-06RA” and “2021-0406-01RA. From 116 individuals in these two clutches of F2 progenies, we successfully obtained phenotype and genotype data. Notably, the F2 progenies exhibited variations in their caudal and dorsal fins (Fig. 2a–h). For each F2 individual, either a single or bifurcated caudal fin was observed, consistent with our previous report (Fig. 2a-–h)^11^. The dorsal-finless phenotypes were also varied, as shown in Fig. 2a–h. Most of the F2 progenies either completely lacked dorsal fin rays, similar to the *Ranchu* strain (Fig. 1b), or the animals had a wild-type-like dorsal fin (Fig. 2a,b,g,h). Nevertheless, some intermediate phenotypes were observed (Fig. 2c–f). For example, some individuals showed a rudimentary dorsal fin, while others had only a few dorsal fin rays (Fig. 2c–f).

To examine the influence of *chdS*^*wt*^ and *chdS*^*E127X*^ alleles on the dorsal fin phenotype, we counted the numbers of dorsal fin rays in the F2 backcross population and genotyped the *chdS* locus in 116 progenies (Fig. 2i,j; Supplementary Tables S1 and S2); the two clutches respectively contributed 67 and 49 individuals to the total. Our analyses of these F2 progenies clearly indicated that the *chdS*^*wt*^ allele influences the dorsal-finless phenotype (Fig. 2a–f, Supplementary Tables S1 and S2). In both clutches, most of the individuals with a *chdS*^*E127X/E127X*^ genotype exhibited bifurcated anal and caudal fins, although many still exhibited single anal and caudal fins (Supplementary Tables S3 and S4). These findings were again consistent with our previous report^11^.

The *chdS*^*E127X/E127X*^ progenies could be categorized as having wild-type and mutant dorsal fins based on the number of the dorsal fin rays present. Our previous research showed that most wild-type goldfish have a total number of dorsal fin rays ranging from 17 to 22 at the late larval stage (Pr stage), so we assigned a mutant phenotype classification to individuals with less than 17 dorsal fin rays in their dorsal fin^16^. Based on this criterion, more than half of the *chdS*^*E127X/E127X*^ progenies exhibited a dorsal fin mutation phenotype (Fig. 2i,j; Supplementary Tables S1–S4). In contrast to the *chdS*^*E127X/E127X*^ individuals, the *chdS*^*wt/E127X*^ F2 progenies tended to show the wild-type phenotype in the dorsal morphology (Fig. 2i,j; Supplementary Tables S1–S4). These results suggested that the presence of *chdS*^*wt*^ significantly suppresses the expression of the dorsal-finless phenotype; nevertheless, several *chdS*^*wt/E127X*^ F2 progenies showed anomalous mutated phenotypes at the dorsal fin (Fig. 2i,j; Supplementary Fig. 2a–d). In short, our data strongly imply that the expression of mutations in the median fins are genetically linked with *chdS* locus.

### mRNA microinjection rescue

**Figure 3:**
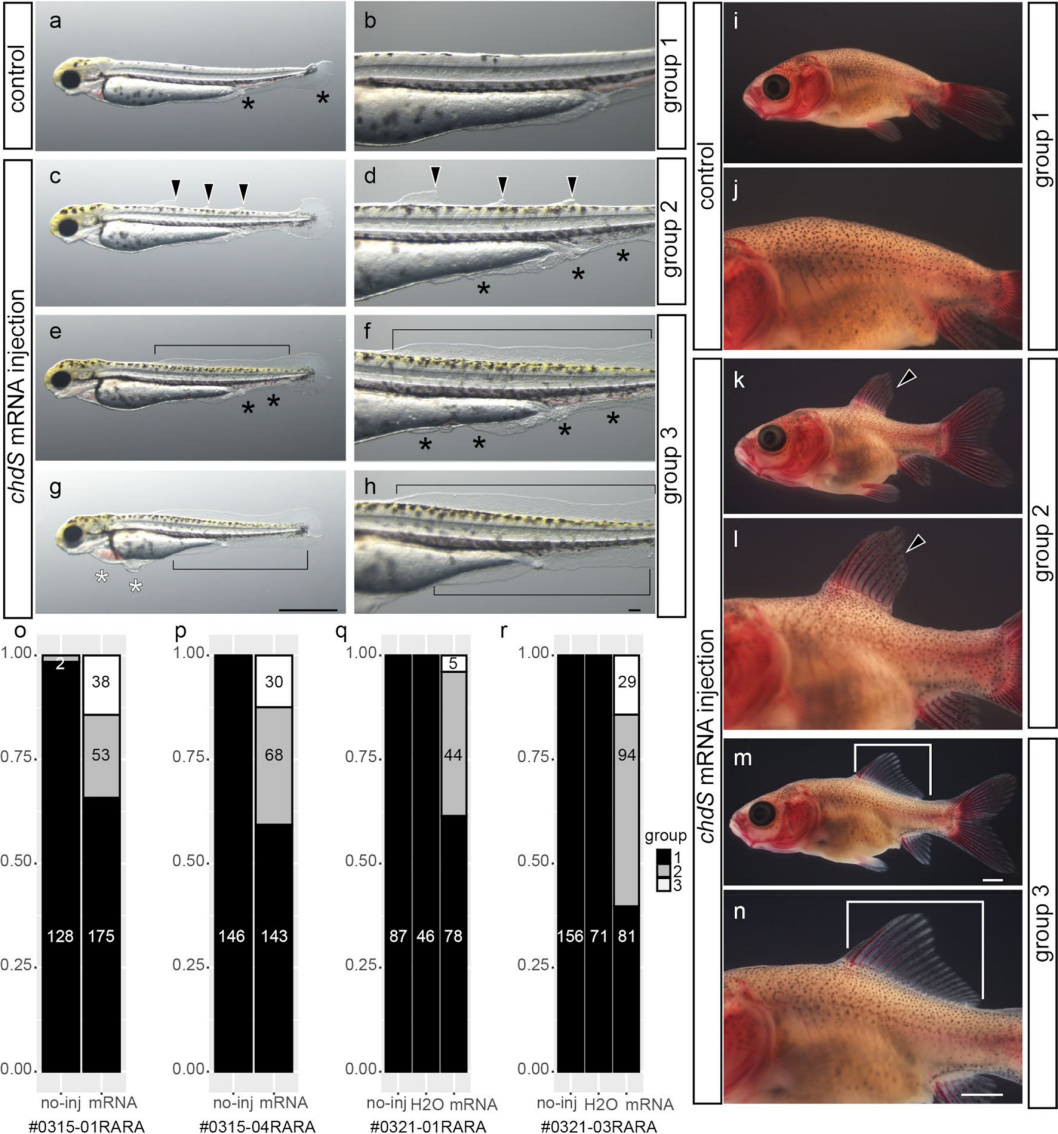
Rescue of the dorsal-finless phenotype by mRNA microinjection. **(a**–**h)** Representative phenotypes of hatched larvae (3dpf). Control (**a**, **b**) or *chdS*^*wt*^ mRNA injected hatched larvae (**c**-**h**) (arrowheads, partially rescued dorsal fin fold; black bracket, completely rescued fin fold; black asterisks, mutated fin fold at the ventral side; white asterisks, malformation of the anterior ventral side tissues). Panels of (**b**, **d**, **f** and **h**) are magnified views of (**a**, **c**, **e**, and **g**), respectively. Hatched larva exhibiting group 1 (**a**, **b**), group 2 (**c**, **d**) or group 3 (**e**–**h**) phenotypes. (**i**–**n**) Late larvae. Control (**i**, **j**) and *chdS*^*wt*^ mRNA injected late larvae (**k**–**n**). Panels of (**j**, **l**) and (**n**) are magnified views of (**i**, **k**), and (**m**), respectively (black arrowhead, partially rescued dorsal-fin; white bracket, completely rescued dorsal fin). Late larvae on panels on (**i**, **k**), and (**m**) are derived from the hatched larvae of group 1, group 2, and group 3, respectively. (**o**-**r**) Proportion of rescued specimens following injection of embryos with the wild type *chdS* mRNA (chi-squire test p < 0.0001 in all clutches). Scale bars = 0.1 mm (**h**), 1 mm (**g**, **m**, **n**). Panels of hatched larvae (**a**, **c**, **e**, **g**), the magnified views of the hatched larvae (**b**, **d**, **f**, **h**), late larvae (**i**, **k**, **m**), and the magnified view of the late larvae (**j**, **l**, **n**) are shown at the same magnification.

To definitively test whether the *chdS* locus influences the expression of the mutation phenotype in the dorsal fin and the other median fins, we designed a rescue experiment in *Ranchu* strain progenies. In this experiment, we performed *chdS* mRNA microinjection, which could rescue the twin-tail phenotype in our previous study^11^. Microinjections of *chdS*^*wt*^ mRNA were made into the fertilized eggs derived from *Ranchu* parents (Methods) (Fig. 1e). The experiment was repeated four times in independent clutches (#2022-0315-01RARA, #2022-0315-04RARA, #2022-0321-01RARA, #2022-0321-03RARA), allowing us to examine the phenotypes in a total of 1474 hatched larvae at 3 days post-fertilization (dpf) (Supplementary Table S5). The examinations focused on the morphologies of the median fin fold and its derivatives (Fig. 3a–n; Supplementary Fig. S2).

Our rescue experiments demonstrated that *chdS*^*wt*^ mRNA microinjected into early-stage larvae caused the expression of different phenotypes than those seen in the control larvae (non-injected or water-injected larvae), in terms of the median fin fold at pre- and post-cloacal levels (Fig. 3a–h). Moreover, these phenotypic variations of the median fin fold were related to the dorsal fin morphology at the late larval stage (Fig. 3i–n). All of the control hatched larvae exhibited a relatively uniform ventralized phenotype (e.g., bifurcated and/or disrupted fin folds at the ventral side and enlarged blood island), as described in *chordin*-depleted teleost species, in addition to a reduced dorsal fin fold from the caudal to mid-trunk level (equivalent of yolk extension region) (Fig. 3a,b)^11,17–19^. On the other hand, the mRNA-injected larvae exhibited various phenotypes (Fig. 3c–h). A number of the hatched larvae exhibited rescue of the median fin fold phenotype at both dorsal and ventral sides after microinjection of the mRNA. This rescue was consistent with our previous mRNA rescue experiment, although several hatched larvae exhibited malformations in the heart and the epithelial tissues at the anterior regions (Fig. 3g,h)^11,20^.

Notably, the level of rescue in the hatched larvae appeared to vary in terms of the dorsal fin fold phenotype (Fig. 3c–h). Based on the dorsal fin morphology, we categorized the control and mRNA-injected larvae into three different groups as follows: (i) no dorsal fin fold at the level of yolk and post-yolk levels (group 1; Fig. 3a,b), (ii) partial dorsal fin fold at the level of yolk and post-yolk levels (group 2; Fig. 3c,d), and (iii) a wild-type equivalent phenotype of complete dorsal fin fold at the yolk and post-yolk levels (group 3; Fig. 3e–h) (for further descriptions of each group, see Methods). The morphological observations of these categorized larvae at the late larval stage (Fig. 3i–n) were entirely consistent with the early larval phenotypes (Fig. 3a–h). More specifically, at the late larval stages, dorsal fin rays were not observed in group 1 individuals, dorsal fin rays were missing at several body levels in group 2 individuals, and dorsal fin rays were completely developed in the group 3 individuals (Fig. 3i–n)^16,21^. This concordance of phenotypes suggested that *chdS*^*wt*^ mRNA at the early embryonic developmental stage influences the eventual skeletal morphology of the dorsal fin (Fig. 3a–n).

To analyze the result of the mRNA microinjection rescue experiment in further detail, we compared the larvae within each clutch. The comparison showed a significant reduction of group 1 individuals among mRNA-injected embryos (Fig. 3o–r). Although the reactivity to the mRNA varied between clutches, we obtained similar results in the four independent experiments (Fig. 3o–r). Based on the consistent results from the mRNA microinjection rescue experiments and the F2 segregant analyses, we conclude that it is highly probable that the dorsal-finless phenotype and its related mutated genes are under the strong influence of the presence/absence of *chdS*^*wt*^ allele. To our knowledge, this is the first study to identify the responsible gene of the dorsal-finless phenotype in ornamental goldfish by applying not only genetic linkage analyses but also functional analyses in goldfish embryos.

## Discussion

**Figure 4:**
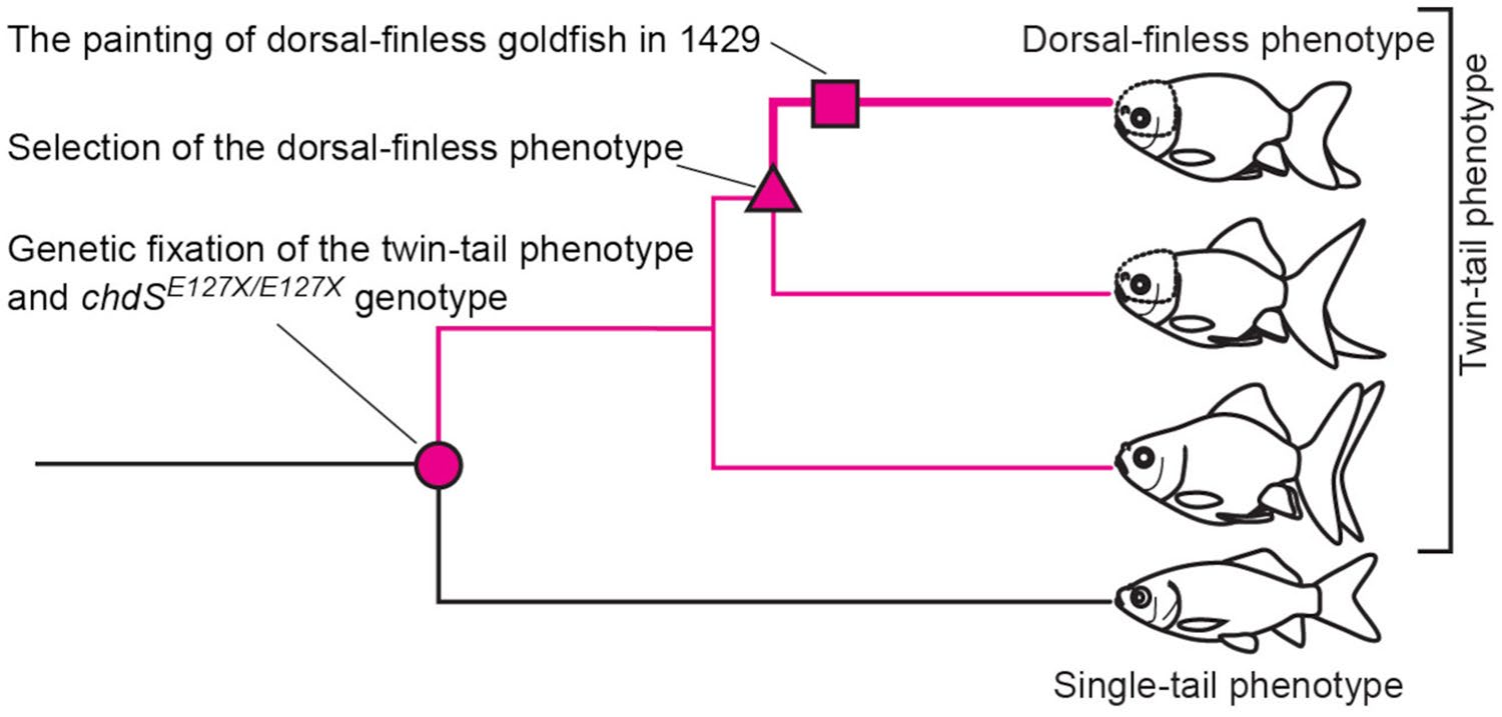
Schematic representation of the appearance of the dorsal-finless phenotype. The topology of the phylogenetic tree is based on the molecular phylogenetic analyses^6,14^. The magenta circle indicates the genetic fixation of the *chdS*^*E127X/E127X*^ genotype in the ornamental goldfish sub-population (twin-tail strains). The magenta triangle indicates the starting point of the selective pressures to dorsal-finless phenotype. The expressivity/penetrance of dorsal-finless phenotype was increased in the *chdS*^*E127X/E127X*^ sub-population (thin magenta line), and this was enhanced in the lineage of the dorsal-finless goldfish strain (bold magenta line) in the phylogenetic tree. The magenta box indicates the record of the first painting of the dorsal-finless phenotype in 1429 CE (please see also Supplementary Information).

In this study, we show that the *chdS* gene is responsible not only for the twin-tail phenotype but also for the dorsal-finless phenotype, suggesting that the *chdS* gene has pleiotropic functions that influence the formation of mutated morphologies in all median fins. Based on our results, we can posit an evolutionary process of the dorsal-finless phenotype. (1) Before the genetic fixation of the twin-tail phenotype, the penetrance/expressivity of the dorsal-finless phenotype was suppressed due to the *chdS*^*wt*^ (magenta circle in Fig. 4). (2) After the genetic fixation of the twin-tail phenotype, the penetrance/expressivity of the dorsal-finless phenotype was increased due to the absence of *chdS*^*wt*^ (magenta lines in Fig. 4). (3) In certain lineages of goldfish (called the “Ranchu group” in Kon and his colleagues^6^), the dorsal-finless phenotype was intensively selected by breeders and fanciers (magenta triangle and bold magenta line in Fig. 4). (4) Finally, the appearance of *Ranchu*-group (dorsal-finless and twin-tail phenotype) goldfish was recorded in an early painting due to its common presence in the breeding population of ornamental goldfish (magenta square in Fig. 4; see Supplementary information). It seems that this hypothetical evolutionary process is completely consistent with the early report by Matsui^22^, which describes the occasional appearance of dorsal fin malformations in the *Ryukin* strain. This report states that although the majority of progenies of this strain show a complete dorsal fin phenotype, 51 of 1222 exhibited a “rudimentary dorsal finned phenotype”, suggesting that the dorsal-finless phenotype can occur in goldfish with the *chdS*^*E127X/E127X*^ genotype. However, such a low penetrance/expressivity of the dorsal-finless phenotype suggests that some additional mutated alleles should be accumulated before stable expression of the dorsal-finless phenotype can occur.

Although our study does not hint at the additional genetic mutations necessary for the dorsal-finless phenotype, our F2 segregant analyses provides important insights into how the *chdS* gene is related with those other loci in terms of phenotype expression. The fact that several F2 progenies with *chdS*^*wt/E127X*^ genotype expressed the dorsal-finless phenotype indicates that there is at least one other responsible locus for the dorsal-finless phenotype independent of the *chdS* gene (Fig. 2i,j: Supplementary Fig. S2a–d). However, our results from the F2 segregant analyses indicated that the majority of mutated alleles responsible for the dorsal-finless phenotype are under the influence of *chdS* gene. Thus, the *chdS* gene appears to be epistatic to most genes responsible for the dorsal-finless phenotype. Taking into consideration the difficulty of detecting epistatic effects in complex human phenotypes using GWAS studies as well as the complicated molecular networks of *chordin*-related genes, it may be extraordinarily difficult to unambiguously identify the genes responsible for the dorsal-finless phenotype with simple application of a GWAS design on the domesticated population of goldfish^6,7,23^.

In fact, there is still no consensus about the responsible genes for the dorsal-finless phenotype, even though two GWAS reports have been published on the dorsal-finless phenotype by two independent research groups^6,7^. One group identified *lrp6* gene as a candidate gene for the dorsal-finless phenotype, while the other group suggested a relationship exists between the mutated phenotype and the *dhfr* gene^6,7^. These inconsistent findings cannot be adequately explained at the level of the molecular mechanism due to a paucity of studies on these genes in goldfish. However, our study suggests that the inconsistent findings from these GWAS reports might be at least partially due to the complex epistatic relationships between the gene regulatory network and the *chordin* gene^18,24–28^. Our study also strongly indicates that further investigations will be required to determine how the early embryonic developmental process relates to the simultaneous occurrence of the twin-tail and dorsal-finless phenotypes^25–27^.

Careful consideration of the embryonic proximity of the dorsal and caudal fins and their modular relationship may provide a deeper understanding of how these two phenotypes may have simultaneously occurred^12,16,21,29,30^. Molecular developmental studies in zebrafish revealed that the mesenchymal cells of the dorsal- and caudal fins are derived from the different levels of somite, but the epithelial cells are connected to each other at the embryonic stage; the epithelial cells form the fin fold and its primordia^31,32^. Based on the close embryonic proximity of epithelial cells, it is naturally presumed that both dorsal- and caudal fins are under the influence of the same molecular mechanisms. In fact, this interpretation allows us to explain why the majority of our F2 backcross progenies consistently exhibited mutant phenotypes in dorsal, anal, and caudal fins (Supplementary Tables S1, S2). Moreover, previous reports on *szl*-depleted goldfish are consistent with this interpretation^18,33^. Depletion of *szl* gene expression in goldfish embryos leads to the dorsal-finless phenotype, which is related to the *chordin* gene^33^. Although the detailed molecular developmental mechanisms of the dorsal fin disappearance are still unknown, it is certain that the depletion of this gene disrupts dorsal–ventral patterning to generate the dorsal-finless phenotype in the goldfish^33^. Thus, it is reasonable to assume that the morphogenesis processes of the dorsal and caudal fin are under the regulation of the same dorsal–ventral patterning molecular developmental mechanism (which involves the *chordin* genes) at the early embryonic stage, even though these fins are separate structures at juvenile and adult stages.

We can rephrase the aforementioned assumption as follows. The *chdS*^*wt*^ allele might have a buffering capacitor function that allows the dorsal–ventral patterning mechanisms to accumulate genetic mutations^25–27^. This assumption allows us to answer why the twin-tail and the dorsal-finless phenotype might appear quasi-simultaneously during the goldfish domestication history. The accumulation of responsible mutations for the dorsal-finless phenotype might have been masked by the *chdS*^*wt*^ allele, and the effects of these mutations could have been unmasked specifically in the twin-tail goldfish lineages. Of course, our present study cannot completely reject the proposition that all of the responsible mutated alleles of the dorsal-finless phenotype had appeared after the fixation of *chdS*^*E127XE127X*^ in the goldfish population. Nevertheless, an evolutionary scenario that includes a buffering capacitor function of the *chdS* gene seems more plausible than a scenario in which de novo genetic mutations for the dorsal-finless phenotype appeared in the lineage of the Ranchu group, as the first scenario could easily explain why the fixation process of the dorsal-finless phenotype in the twin-tail ornamental goldfish as so rapid (Fig. 4).

Researchers have investigated how masked genetic variations (or cryptic genetic variations) contribute to the expression of phenotypes upon genetic perturbation in several vertebrate species, including teleost species^34–37^. For example, the effects of chaperone protein HSP90 have been investigated in zebrafish and cavefish^34,35^. Moreover, the *mef2ca* gene was also examined in terms of its buffering function in the context of dermal skeleton morphogenesis^36^. Unlike these experimentally derived phenotypic variations, the phenotypic variations in goldfish were established through a process of artificial selection for the stabilization of visually classifiable strains (for example, *Ryukin* and *Ranchu* strains) after the fixation of the *chdS*^*E127X*^ allele^11^. Thus, it is reasonable to assume that the ornamental morphologies of established goldfish strains are the consequence of the canalization of morphogenesis^38–40^. More specifically, the stable expression of both the twin-tail and dorsal-finless phenotypes has been canalized in the lineage of the *Ranchu* strain, but not in *Ryukin* strain. This fact would imply that the various dorsal-finless phenotype-associated candidate genes identified by GWAS analyses might actually contribute to the phenotypic canalization, rather than acting as decisive factors for the dorsal-finless phenotype^6,7^. We hope that future studies may combine the GWAS approach with molecular developmental genetics methodologies to better reveal the relationships between drastic morphological changes, developmental robustness, and genetic variations in different ornamental goldfish strains.

## Methods

### Goldfish strains

Goldfish were purchased from an aquarium fish agency in Taiwan. Two strains were used in this research, the single tail common goldfish (wild-type) and the *Ranchu* strain. To avoid confusion about the definition of “wild-type,” which may arise from differences in goldfish nomenclature systems used by breeders and researchers, this study defined goldfish individuals with a slender body and a single fin as having a wild-type phenotype (Fig. 1a)^12^. The *Ranchu* strain individuals lack a dorsal fin and exhibit a bifurcated caudal fin (Fig. 1b). The F0 parents were genotyped by PCR and restriction enzyme digestion as described in a previous paper^11^. The wild-type goldfish parents were homozygous for the *chdS*^*wt*^ allele, and dorsal-finless goldfish were homozygous for the *chdS*^*E127X*^ allele. By crossing these parental strains, F1 individuals were obtained (Fig. 1c). These F1 individuals were subsequently used to obtain F2 segregants. The research was performed in accordance with internationally recognized guidelines and ARRIVE guidelines. Ethical approval was from the Institutional Animal Care & Utilization Committee of Academia Sinica, Taiwan (Protocol ID: 19-11-1351).

### Artificial fertilization

The procedure for artificial fertilization was based on our previous report^30^. In the spawning season (March to June), sperm were taken from males and preserved in Modified Kurokura’s extender 2 solution at 4 °C^41^. Eggs were squeezed out from mature females into Teflon-coated dishes. Artificial fertilization was performed using dry methods. The fertilized eggs were spread onto 9-cm Petri dishes, containing tap water (approx. 24 °C).

### Genotyping

The genotype of *chdS* locus was examined by PCR and restriction enzyme digestion as previously reported^11^. PCR primers were designed to amplify the region containing both the *chdS*^*E127X*^ allele and the closely linked AvaI restriction enzyme site. PCR fragments amplified by these specific primers were digested by AvaI, and separated on 2% agarose gels. Genotypes were determined on the basis of the resulting band patterns.

### mRNA microinjection rescue experiment

The pCS2 + plasmid vector containing the coding regions of the *chdS*^*wt*^ sequence were used to synthesize mRNA for the microinjection rescue experiments^42^. The sequence containing the coding region of *chdS*^*wt*^ was amplified by PCR reaction, using a forward primer Sp6 (5′-ATTTAGGTGACACTATAGA-3′) and reverse primer M13R (5′-TCACACAGGAAACAGCTA TGAC-3′). The PCR product was cleaned with a GeneMark DNA Clean/Extraction kit before being used as the DNA template to synthesize capped mRNA. The mRNA synthesis was performed with the mMESSAGE mMACHINE SP6 kit, according to the manufacturer’s instructions (Ambion Inc.). Then, the synthesized mRNA transcripts were purified using a Monarch RNA Cleanup kit and resuspended in nuclease-free water. The microinjection mixture contained 1 μl Phenol Red (Sigma), 1 μl of 2 M KCl, 4.7 μl nuclease-free water, and 3.3 μl synthesized mRNA (300 ng/μl) in a total of 10 μl. Phenol red (Sigma) was used as an indicator at a final concentration of 0.05%. Injection needles were prepared from borosilicate glass filaments (Sutter instrument, BF100-50-10) using a micropipette puller (Model P-1000, Sutter Instrument). A microinjector (Eppendorf Femtojet; Eppendorf) was used to inject mRNA into the center of the yolk of fertilized eggs at the 1–2 cell stage, which were maintained on Petri dishes. Each embryo was injected with 2 μl of the mRNA mixture containing a total of 250 pg mRNA. The injected embryos were then incubated at 24 °C until phenotyping at 3 dpf. Four independent rescue experiments were performed by injecting *chdS*^*wt*^ mRNA into the embryos derived from *Ranchu* strain parents. At 3 dpf, embryos were categorized into three groups (described below). These categorized 3 dpf larvae were maintained for more than one month and assessed for their phenotype at hatching. To examine the potential influence of the mechanical contact with the injection needle, nuclease-free water was injected into the embryos as a control. As expected, mechanical contact did not cause significant changes to the morphology of the larvae during the developmental process.

### Phenotype analysis

The F2 segregants were phenotyped at both late embryonic (3 dpf) and post-embryonic stages. The embryos and larvae were observed under stereomicroscopy. The 3 dpf embryos were categorized into three groups (group 1, group 2 and group 3) based on the position of rescued fin fold shape and its relative position to yolk; the position of dorsal fin rays at the juvenile stage is known to be related with that of the cloaca^16,21^. The criteria for these categories were as follows: group 1 individuals had no dorsal fin fold at the level of yolk; group 2 had dorsal fin fold partially at the level of yolk; and group 3 had a complete dorsal fin fold at the yolk and post-yolk levels. The phenotyping of late larvae and juveniles was conducted based on the numbers of dorsal fin rays. Maintenance of larvae followed previous reports^12,21^. The progenies from Pelvic fin ray stage to juvenile stage were fixed with PFA (4 wt% of paraformaldehyde in 1 × PBS solution) overnight, washed in 70% ethanol, and stained with alizarin red solution (0.02% alizarin red in 70% ethanol). Alizarin red-stained specimens were washed with 70% ethanol to reduce background. Phenotypic observations in larvae and stained specimens were made under stereomicroscopy (SZX16 and SZ16, Olympus). Images were acquired using a stereomicroscope system with digital microscope camera (SZX16 with DP80; Olympus). All data comparisons were made with the R statistical computing package of RStudio (Build 554, version 2022.07.1 + 554).

## Supplementary Information

Supplementary Information.

## Data Availability

The dataset supporting the conclusion of this article are included within the article (Supplementary Tables S1–S5).

## References

[1] Lindblad-Toh K (2005). Genome sequence, comparative analysis and haplotype structure of the domestic dog. Nature.

[2] Rohner N (2009). Duplication of fgfr1 permits Fgf signaling to serve as a target for selection during domestication. Curr. Biol..

[3] Akey JM (2010). Tracking footprints of artificial selection in the dog genome. Proc. Natl. Acad. Sci. U. S. A..

[4] Shapiro MD (2013). Genomic diversity and evolution of the head crest in the rock pigeon. Science.

[5] Schoenebeck JJ, Ostrander EA (2014). Insights into morphology and disease from the dog genome project. Annu. Rev. Cell Dev. Biol..

[6] Kon T (2020). The genetic basis of morphological diversity in domesticated goldfish. Curr. Biol..

[7] Chen D (2020). The evolutionary origin and domestication history of goldfish (*Carassius auratus*). Proc. Natl. Acad. Sci..

[8] Darwin, C. *The Variation of Animals and Plants Under Domestication by Charles Darwin: 1*, Vol. 2 (J. Murray, 1868).

[9] Watase S (1887). On the caudal and anal fins of gold-fishes. J. Coll. Sci. Imp. Univ. Jpn..

[10] Bateson W (1894). Materials for the Study of Variation Treated With Especial Regard to Discontinuity in the Origin of Species.

[11] Abe G (2014). The origin of the bifurcated axial skeletal system in the twin-tail goldfish. Nat. Commun..

[12] Ota KG (2021). Goldfish Development and Evolution.

[13] Nelson JS, Grande TC, Wilson MV (2016). Fishes of the World.

[14] Komiyama T (2009). An evolutionary origin and selection process of goldfish. Gene.

[15] Smartt J (2001). Goldfish Varieties and Genetics: Handbook for Breeders.

[16] Li IJ, Chang CJ, Liu SC, Abe G, Ota KG (2015). Postembryonic staging of wild-type goldfish, with brief reference to skeletal systems. Dev. Dyn..

[17] Fisher S, Halpern ME (1999). Patterning the zebrafish axial skeleton requires early chordin function. Nat. Genet..

[18] Yabe T (2003). Ogon/Secreted Frizzled functions as a negative feedback regulator of Bmp signaling. Dev. Camb. Engl..

[19] Shimada A (2013). Trunk exoskeleton in teleosts is mesodermal in origin. Nat. Commun..

[20] Abe G, Ota KG (2016). Evolutionary developmental transition from median to paired morphology of vertebrate fins: Perspectives from twin-tail goldfish. Dev. Biol..

[21] Li I-J, Lee S-H, Abe G, Ota KG (2019). Embryonic and post-embryonic development of the ornamental twin-tail goldfish. Dev. Dyn..

[22] Matsui Y (1935). Fukkokuban, Kagakuto Shumikaramita Kingyono Kenkyuu.

[23] Tam V (2019). Benefits and limitations of genome-wide association studies. Nat. Rev. Genet..

[24] Muraoka O (2006). Sizzled controls dorso-ventral polarity by repressing cleavage of the Chordin protein. Nat. Cell Biol..

[25] Plouhinec J-L, Zakin L, Moriyama Y, De Robertis EM (2013). Chordin forms a self-organizing morphogen gradient in the extracellular space between ectoderm and mesoderm in the Xenopus embryo. Proc. Natl. Acad. Sci..

[26] De Robertis EM, Tejeda-Muñoz N (2022). Evo-devo of urbilateria and its larval forms. Dev. Biol..

[27] Inomata H, Haraguchi T, Sasai Y (2008). Robust stability of the embryonic axial pattern requires a secreted scaffold for chordin degradation. Cell.

[28] Langdon YG, Mullins MC (2011). Maternal and zygotic control of zebrafish dorsoventral axial patterning. Annu. Rev. Genet..

[29] Abe G, Ide H, Tamura K (2007). Function of FGF signaling in the developmental process of the median fin fold in zebrafish. Dev. Biol..

[30] Tsai H-Y, Chang M, Liu S-C, Abe G, Ota KG (2013). Embryonic development of goldfish (*Carassius auratus*): a model for the study of evolutionary change in developmental mechanisms by artificial selection. Dev. Dyn.

[31] Parichy DM, Elizondo MR, Mills MG, Gordon TN, Engeszer RE (2009). Normal table of postembryonic zebrafish development: Staging by externally visible anatomy of the living fish. Dev. Dyn..

[32] Miyamoto K, Kawakami K, Tamura K, Abe G (2022). Developmental independence of median fins from the larval fin fold revises their evolutionary origin. Sci. Rep..

[33] Abe G, Lee S-H, Li I-J, Ota KG (2018). An alternative evolutionary pathway for the twin-tail goldfish via szl gene mutation. J. Exp. Zool. B Mol. Dev. Evol..

[34] Yeyati PL, Bancewicz RM, Maule J, Van Heyningen V (2007). Hsp90 selectively modulates phenotype in vertebrate development. PLoS Genet..

[35] Rohner N (2013). Cryptic variation in morphological evolution: HSP90 as a capacitor for loss of eyes in cavefish. Science.

[36] DeLaurier A (2014). Role of mef2ca in developmental buffering of the zebrafish larval hyoid dermal skeleton. Dev. Biol..

[37] Green RM (2017). Developmental nonlinearity drives phenotypic robustness. Nat. Commun..

[38] Waddington CH (1942). Canalization of development and the inheritance of acquired characters. Nature.

[39] Wagner GP, Booth G, Bagheri-Chaichian H (1997). A population genetic theory of canalization. Evolution.

[40] Hallgrimsson B (2019). The developmental-genetics of canalization. Seminars in Cell & Developmental Biology.

[41] Magyary I, Urbanyi B, Horvath L (1996). Cryopreservation of common carp (*Cyprinus carpio* L.) sperm II. Optimal conditions for fertilization. J. Appl. Ichthyol..

[42] Rupp RAW, Snider L, Weintraub H (1994). Xenopus embryos regulate the nuclear localization of XMyoD. Genes Dev..

