# Supplementary material for: Pleiotropic functions of chordin gene causing drastic morphological changes in ornamental goldfish
Source: Sci Rep. 2022 Nov 19;12:19961. doi: 10.1038/s41598-022-24444-7 (PMC9675773; doi:10.1038/s41598-022-24444-7)
Supplement: Supplementary file 1 — Supplementary Information. [file 41598_2022_24444_MOESM1_ESM.pdf]

Supplementary information

Supplementary Note

The origin of the dorsal-finless goldfish was described as the part of "History of goldfish domestication" in Supplementary Text of Chen et al., (2020)[7]. Although their description is the most updated and consistent with the historical description to our knowledge, there are several points of confusion that arise from references relating with the dorsal-finless goldfish, nomenclature of morphotypes and strain names of goldfish. To avoid compounding these issues, we provide several descriptions herein. Although Chen et al. (2020)[7] cited Matsui (1981)[43] as a source of evidence that the dorsal-finless phenotype appeared in 1429, we were unable to find the corresponding descriptions in Matsui (1981)[43]. At least in our survey of the book, we could not identify any explicit descriptions about the year of the painting under examination. Nevertheless, the oldest painting of dorsal-finless ornamental goldfish by The Xuande Emperor Zhu Zhanji (朱瞻基) in 1429 is archived as seven photographic plates at Tokyo National Research Institute for Culture Properties. The name of the picture is "Fish and Weeds (魚藻図)". The original photographic plates are numbered ID12397-12402 and accessible at the institute's website (https://www.tobunken.go.jp/archives/, accessed Sept. 28, 2022). Moreover, a fancier of goldfish has posted an introduction of the drawn goldfish in this painting online (https://www.youtube.com/watch?v=3wTMmibqgDI, accessed Sept. 19, 2022). Importantly, all goldfish with their entire bodies shown in the painting lack the dorsal fin and had twin-tail phenotype. Written sources of goldfish morphological mutations have stated that the twin-tail phenotype was first described in the 1500s (Chen 1956)[44]. However, it seems clear that at least one dorsal-finless strain with the twin-tail phenotype had already appeared in 1429.

Supplementary References

43. Matsui, Y., Kumagai, T. & Betts, L. C. *Pet Library Goldfish Guide*. (Pet Library Limited, 1981).

44. Chen, S. C. A history of the domestication and the factors of the varietal formation of the common goldfish, *Carassius auratus*. *Sci. Sin.* **5**, 287–321 (1956).

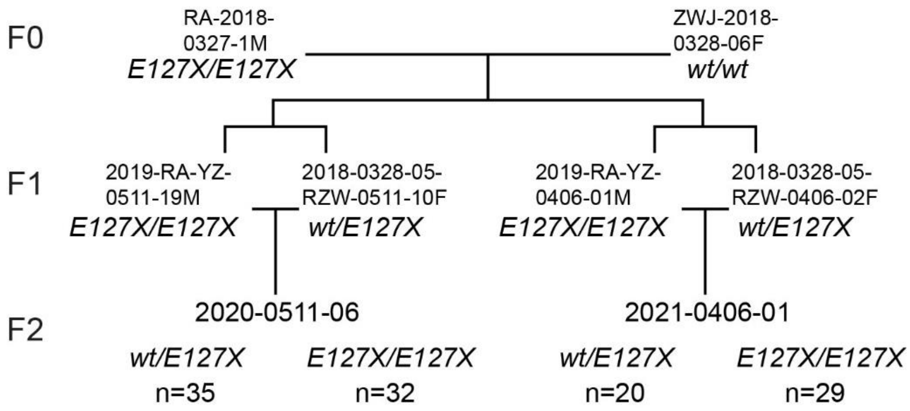

Supplementary Fig. S1: Pedigree of the parents.

The small and large letters indicate the strain name and genotypes, respectively. The handling codes of ZWJ, RA, and RZW respectively indicate the single-tail common goldfish, *Ranchu*, and hybrid of the single-tail common goldfish and *Ranchu* strains.

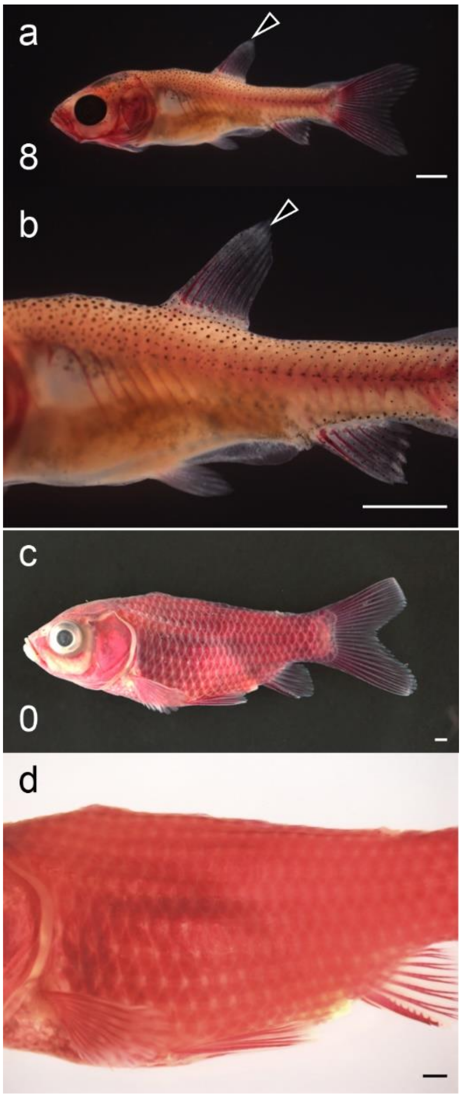

Supplementary Fig. S2: Dorsal-finless mutation in the single-tail phenotype goldfish.

Lateral views of F2 progenies. The genotype of these individuals is *chdS*$^{wt/E127X}$. The individuals in panels **a** and **c** respectively have a reduced number of dorsal fin rays and exhibit the dorsal-finless phenotype. Panels **b** and **d** are magnified views of **a** and **c**, respectively. The numbers at the lower left corner of panels of **a** and **c** indicate the numbers of dorsal fin rays. Scale bars are approximately 1 mm.

Supplementary Table S1: *chdS* genotype and dorsal fin in clutch #2020-0511-06 RA

dorsal fin ray number	*wt/E127X*	*E127X/E127X*	total
less than 17	12	29	41
more than 17	23	3	26
total	35	32	67

Chi-square: $p<10^{-10}$

Supplementary Table S2: *chdS* genotype and dorsal fin in clutch #2021-0406-01 RA

dorsal fin ray #	*wt/E127X*	*E127X/E127X*	total
less than 17	1	19	20
more than 17	19	10	29
total	20	29	49

Chi-square: p<0.0005

Supplementary Table S3: The median fins of the *E127X/E127X* goldfish (#2020-0511-06)

dorsal fin ray #	single anal/caudal fin	bifurcated anal/caudal fin	total
less than 17	5	24	29
more than 17	1	2	3
total	6	26	32

Fisher’s exact test p=0.4758

Supplementary Table S4: The median fins of the *E127X/E127*X goldfish (#2021-0406-01)

dorsal fin ray #	single anal/caudal fin	bifurcated anal/caudal fin	total
less than 17	0	0	20
more than 17	3	3	6
total	3	3	26

Fisher’s exact test p=0.023

Supplementary Table S5: Microinjection rescue experiments

clutch ID	Injected reagents	group 1 phenotype	group 2 phenotype	group 3 phenotype	total
2022-0315-01-RARA	no injection	0	2	128	130
	250 pg of chdA mRNA	38	53	175	266
2022-0315-04-RARA	no injection	0	0	146	146
	250 pg of chdA mRNA	30	68	143	241
2022-0321-01-RARA	no injection	87	0	0	87
	H2O	46	0	0	46
	250pg of chdS mRNA	78	44	5	127
2022-0321-03-RARA	no injection	156	0	0	156
	H2O	71	0	0	71
	250pg of chdS mRNA	81	94	29	204
total		587	261	26	1474
